# The Development of a New Phosphogypsum-Based Construction Material: A Study of the Physicochemical, Mechanical and Thermal Characteristics

**DOI:** 10.3390/ma14237369

**Published:** 2021-12-01

**Authors:** Hela Garbaya, Abderraouf Jraba, Mohamed Amine Khadimallah, Elimame Elaloui

**Affiliations:** 1 Laboratory of Materials Applications in Environment, Water and Energy, Faculty of Sciences, University of Gafsa, Gafsa 6029, Tunisia; helagarbaya@hotmail.fr (H.G.); jrabaraoof@gmail.com (A.J.); limamealoui@gmail.com (E.E.); 2 Faculty of Sciences of Gabes, University of Gabes, Gabes 6029, Tunisia; 3 Civil Engineering Department, College of Engineering, Prince Sattam Bin Abdulaziz University, Al-Kharj 16273, Saudi Arabia

**Keywords:** phosphogypsum, valorization, construction material, mechanical and thermal properties

## Abstract

Phosphogypsum (PG) is a waste (or by-product) of the production of phosphoric acid, a basic constituent in the manufacturing of modern fertilizers. The annual production of phosphogypsum in Tunisia is currently estimated to be 10 million tons. Its storage in slag in close proximity to production plants generates pollution problems; however, valorization may be a solution. The present paper proposes a simple process for the valorization of this by-product into a construction material. Several physicochemical characterizations are used to prove the characteristics of samples. The chemical composition shows that PG is a gypsum compound with several impurities. The morphological analyses show that the powder materials are mesoporous with a lower specific area. The structural characterizations show that these solids play the role of a water pump as the degree of hydration changes from 2 to 0 and vice versa, depending on the temperature. Mechanical and thermal analyses show that the prepared formulation is brittle and insulating, which presents opportunities for it to be used as a decoration material.

## 1. Introduction

Phosphogypsum (PG) represents the majority of the solid waste produced by the phosphate industry. Phosphoric acid and calcium sulfate dehydrate phosphogypsum are produced by the decomposition of extracted raw phosphate rocks with concentrated sulfuric acid at a temperature range of 75–80 °C. The chemical reaction of PG production is [1]:

$${\left(Ca_{3}{\left(PO_{4}\right)}_{2}\right)}_{3}\mathrm{Ca}F_{2}+10H_{2}SO_{4}+20H_{2}O\underset{\;}{\to }\;6H_{3}PO_{4}+10\mathrm{CaS}O_{4}.2H_{2}O+2\mathrm{HF}$$

Phosphoric acid is mainly used in the production of phosphorus fertilizers such as DAP (diammonium phosphate) and MAP (monoammonium phosphate). For every ton of P_2_O_5_ produced as phosphoric acid, five tons of dry mass phosphogypsum are produced. This equates to an annual quantity of waste of 10 million tons. Phosphogypsum is usually deposited in large stockpiles without any treatment [2,3,4].

For decades, the only way of recycling PG was by using it as an additive in agriculture. Mesic et al. demonstrated the positive effects of PG for soil, water and plants [5]. Waste PG is used mainly in agriculture with several methods of recycling for the fertilization and amelioration of soil rentability. It is used as a fertilizer in agriculture because of its high volumes of calcium, phosphorus and sulfur [6].

Many studies have suggested that phosphogypsum can be used as a substitute for natural gypsum to control the hydration reaction rate in Portland cement production [7]. PG has also been used in the manufacturing of bricks: the incorporation of 30% of PG into annealed clay bricks provides a product that successfully satisfies the standard requirements [8].

In 2021, Ajam [9] proved that the use of Tunisian PG in non-load-bearing brick fabrication requires a low amount of energy and consumes a large amount of waste, which largely reduces environmental pollution, in addition to the high socioeconomic benefits. In addition, this study shows that the radioactive emission of the components of this brick is below the limit values recommended by the standards, and therefore its use is safe. Moreover, Hamdi et al. 2020 [10] prove that Tunisian PG have potential uptake in the material construction industry as paving blocks.

Despite the recycling routes presented above, the large quantities of phosphogypsum generated pose a problem of space especially in urban areas. Over 85% of PG is stored in close proximity to phosphoric acid production units in stockpiles that can reach tens of meters in height. The remaining 15% is either reused or thrown into the sea [11,12]. This management of PG waste presents an extreme threat to human and marine life. The study by Rouis et al. [13] shows that phosphogypsum is a by-product that harms the environment if not stored properly; the storage and recycling of phosphogypsum present the main challenges of the phosphate industry in many countries [8].

In light of the above, the objective of the present paper is presented in four parts. The first part is the study of the radioactivity of generated phosphogypsum, aiming to ensure that PG is safe to be used as construction material. The second part examines the physicochemical characteristics of raw, washed and treated PG as well as prepared samples (PGM) using FTIR, XRD, SEM, EDX, adsorption/desorption of N_2_ at 77 K and XRF analyses. The last section discusses the mechanical and thermal behaviors of the prepared formulation.

## 2. PG Radioactivity

Usually, PG contains radioactive elements. The radioactivity of PG (in particular (α)) is due to the radium content resulting from the decomposition of uranium (present in the phosphate ore). To ensure that the use of PG as a construction material does not pose any danger to users, we have chosen the study of the radioactivity of two types of PG (PG from Croatia and PG from Tunisia).

The activities and concentrations of the different radionuclides in Croatia PG (Lonjsko Polje Nature Park) [14] are presented in Table 1. The major sources of radioactivity in PG are ^238^U and ^232^Th [15]. Uranium is the main environmental radiotoxic element associated with phosphoric acid production; it is transferred from a non-mobile fraction in the phosphate rock to a bioavailable fraction in phosphogypsum [16].

The radiation dose resulting from phosphogypsum piles or received by workers is negligible compared with the average annual effective dose from natural sources [17]. The resulting radiation dose caused by phosphogypsum used as a construction or plaster material can be considered to be negligible [18,19].

Sfar et al. [20] measured the activity of natural radioelements in three Tunisian PGs with different storage times using gamma spectrometry. They noted a decreasing trend of the concentrations of ^238^U and ^232^Th from the most recent to the oldest phosphogypsum, respectively (Table 2). This reduction was most likely due to leaching by natural processes, mainly rainwater. The concentration of ^226^Ra in phosphogypsum remained constant during storage. The measurements of thorium confirmed that ^232^Th preferentially passed into phosphoric acid during the manufacturing process [20].

On the basis of the previously cited studies, it can be concluded that phosphogypsum does not exhibit any nuclear activity that is harmful to humans or to the environment. As a result, its recovery as a construction or insulation material presents a solution for environmental decontamination and not a new danger.

## 3. Experimental Procedure

### 3.1. Sample Preparation

**Figure 1:**
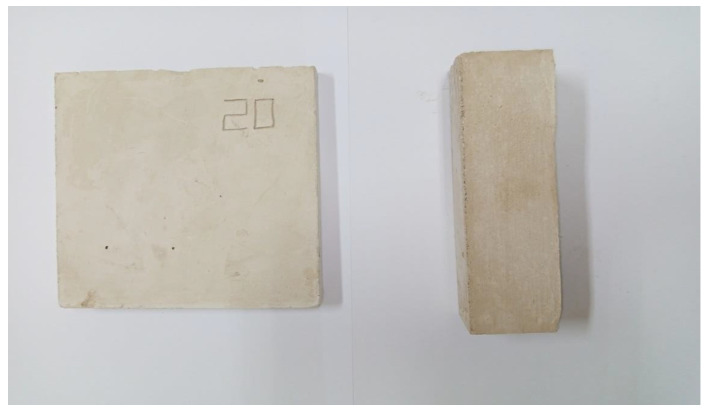
Geometric forms of the PGM 1/1 and PGM 1/2 samples.

Wet natural phosphogypsum (PGF) was directly obtained from the slag heap of the Tunisian Chemical Group M’dhilla Plant (Gafsa, Tunisia). The washed phosphogypsum (PGW) was obtained by washing the PGF; this was placed in a large sieve and washed several times until the wash water obtained a neutral pH (between 6.7 and 7). The PGW was left to dry in the open air for 7 days. The dried PGW was ground by a mechanical grinder equipped with an 0.5 mm sieve to obtain a fine and uniform powder. The PGT was obtained by the thermal treatment of the PGW powder at 200 °C for 12 h [14]. The PGM 1/1 and PGM 1/2 samples were prepared by mixing PGT with water at a PGT/water ratio of 1:1 and 1:2, respectively. The mixture was then poured into molds as needed. The geometric dimensions of the prepared materials were 4 × 4 × 1 cm for the thermal test and 4 × 4 × 16 cm for the mechanical test (Figure 1).

### 3.2. Characterization

The characterization of the phosphogypsum samples was measured by different techniques. The morphological analysis was determined by environmental scanning electron microscopy (SEM) (Quanta 200-FEI) with an accelerating voltage of 15 kV coupled to an EDAX probe. The textural analysis was obtained using a micromeritics instrument (model ASAP 2020 V4.03). The porosity and specific surface area were measured at 77 °K after degassing for 4 h at 105 °C under a vacuum (10 µm/Hg). The FTIR spectra were recorded in KBr pellets using a Shimadzu S400 instrument. The spectra of the solids were obtained using KBr pellets. Prior to the measurements, PG and KBr were mixed at a quality ratio of 1:100. The vibrational transition frequencies were reported in transmittance versus the wave numbers (cm^−1^). The structural properties of the samples were determined using a PANanalytical X’Pert Pro wide-angle X-ray powder diffractometer equipped with a copper anticathode that produced 15,418 Å Cu Kα radiation. An X-ray fluorescence (XRF) Philips sequential wavelength dispersion unit (model PW-1404) was used to determine the elemental composition. The mechanical properties were obtained using a universal ZWICK/ROELL machine. The test bench was equipped with self-tightening jaws and a force cell with a capacity of 5 kN. It was controlled by TEST EXPERT software, which logged the test parameters, acquired, and processed the data. The thermal conductivity and diffusivity coefficient measurements were obtained using a Hot-Disc TP 2500 apparatus. A probe (reference 5465) with a radius of 3189 mm was used. A heating power of 80 mW was applied for 20 s. Further details concerning this method are available in [21,22,23,24,25].

## 4. Results and Discussion

### 4.1. X-ray Fluorescence

As seen in Table 3, the phosphogypsum in these various states was formed mainly by gypsum (CaSO_4_) with the presence of other elements with a low percentage such as Si, Ti, Na, Mg and Fe. The presence of these elements could be attributed to the ore of the phosphate used in the industrial process. This composition was similar to that cited by Mechi et al. [26]. The treatment of PGF did not significantly affect the chemical composition but it increased the CaO/SO_3_ ratio from 0.83 to 0.95.

### 4.2. Powder X-ray Diffraction

**Figure 2:**
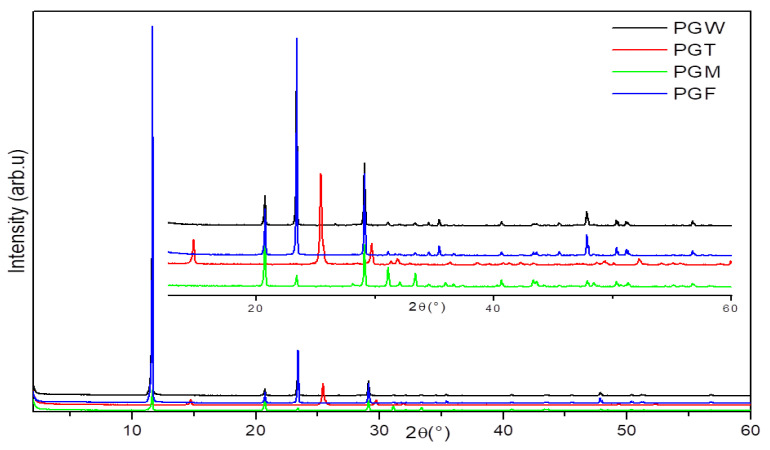
X-ray diffractograms of the PGF, PGW, PGT and PGM samples.

The registered X-ray diffractograms of the different phosphogypsum samples studied showed only the presence of the characteristic peaks of CaSO_4_. These results confirmed the gypsum aspect of PG observed by XRF. The characteristic peaks of the different hydration degrees of the gypsum (CaSO_4_, CaSO_4_.½H_2_O and CaSO_4_.2H_2_O) appeared. Figure 2 indicates that only the characteristic peaks of anhydrous CaSO_4_ appeared in the spectrum of PGT; the lack of coordinate water in this sample could be explained by the thermal treatment at 200 °C. The diffractograms of the other two samples (PGF and PGW) showed the presence of two degrees of hydration (2H_2_O and ½H_2_O). The presence of these degrees of hydration was because they were mixed with water both from the industrial process for PGF and from the washing water for PGW. During the process of preparing PG for use as a building and/or decoration material, phosphogypsum changed from a hydration level of ½ and 2 (PGF and PGW, respectively) to an anhydrous state of 0 (PGT). In its finished state, the prepared material sample (PGM) regained the hydration levels of ½ and 2 (Figure 2); this was due to being mixed with water and the low temperature of the treatment (T = 40 °C).

**Figure 3:**
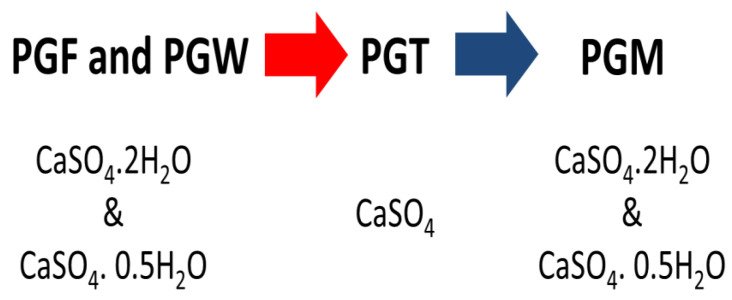
Hydration–dehydration of PG.

It should be noted that the PG with these different degrees of hydration presented a hydration–dehydration phenomenon. The PG in its natural or washed form (PGF, PGW) was gypsum hydrated at ½ and/or 2 H_2_O. After a thermal treatment at 200 °C, it became anhydrous (CaSO_4_) and it obtained the degrees of ½ and/or 2 H_2_O by being mixed with water (PGM). This reversible hydration–dehydration phenomenon that the PG presented in its different states allowed us to qualify it as water pump, as shown in Figure 3.

### 4.3. FTIR Analysis

**Figure 4:**
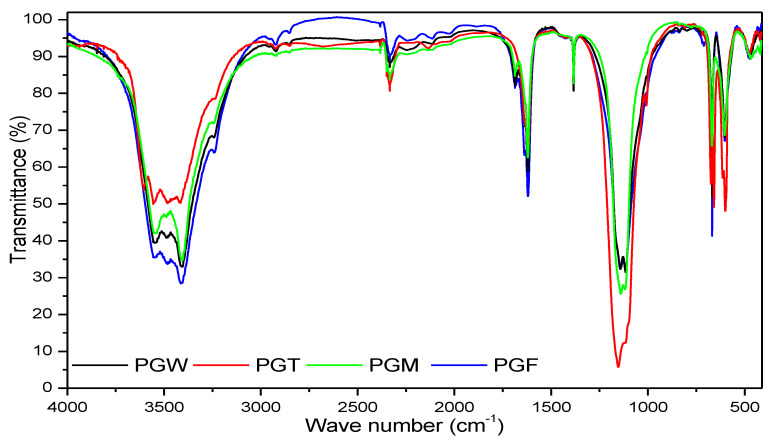
FTIR spectra of the PGF, PGW, PGT and PGM samples.4.4. BET Analysis.

**Figure 5:**
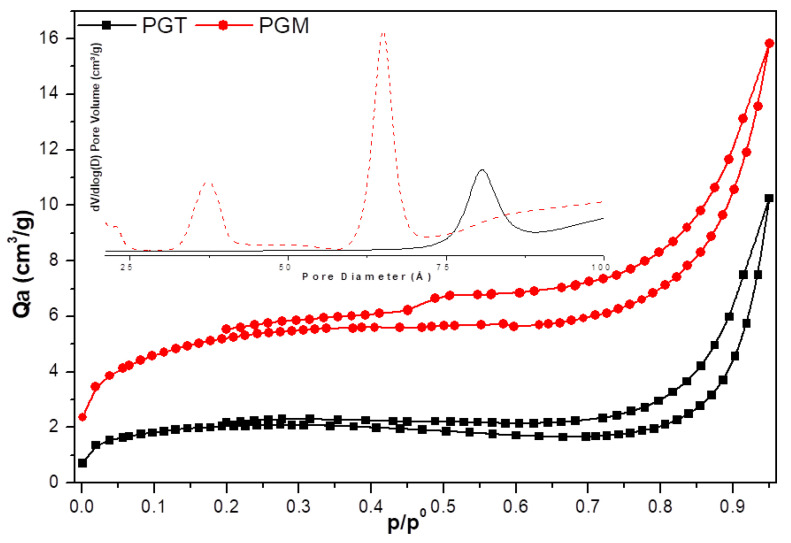
Nitrogen adsorption/desorption isotherms and the pore distribution of the PGT and PGM samples.

**Figure 6:**
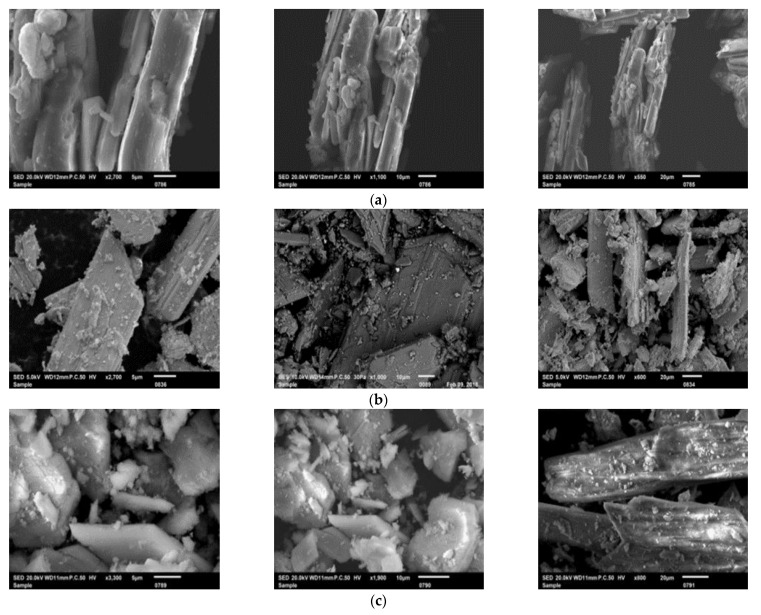

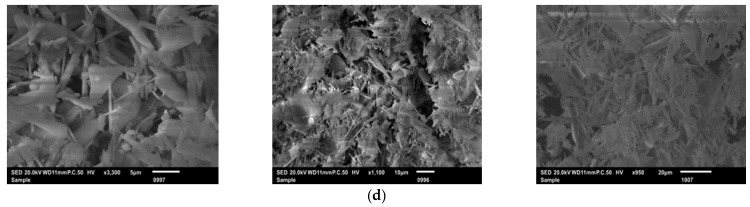
SEM micrographs of: PGF (**a**), PGW (**b**), PGT (**c**) and PGM (**d**).

The FTIR spectra of the PG in the different states (Figure 4) confirmed two results (one qualitative and the other quantitative) shown by the X-ray diffractograms, namely, that the PG was mainly composed of gypsum. This was represented by the peaks of SO_4_^2−^ lying at 594 cm^−1^, 1099 cm^−1^ and 2132 cm^−1^ [26,27,28], as shown in Figure 5 and Figure 6. The second result consisted of the quantitative variation in the peaks of the water at 1620 cm^−1^, which was attributed to the vibrations of the OH groups of the water. At 3590 cm^−1^, this corresponded with the elongation of the internal OH groups [29,30]. The spectrum of PGT presented the least important peaks compared with those of the states mixed with water.

The resulting isotherms of the surface analysis of the treated and modeled phosphogypsum (Figure 5) were type IV, which corresponded with mesoporous solids [31]. This isotherm type corresponded with multimolecular adsorption or a gradual increase in the adsorbed layer thickness. The presence of a type B hysteresis curve was characteristic of slot-shaped porosities [31].

The studied samples could be classified as a mesoporous solid with a low specific surface area of 6.7 m^2^/g for PGT and 17.5 m^2^/g for PGM (Table 4). The pore distribution of these two samples, as presented in Table 4, confirmed the mesoporous properties of the materials with an average pore size of 10.9 nm for PGM and 18.1 nm for PGT. The decrease in the pore size from 18.1 nm for PGT to 10.9 nm for PGM was responsible for the increase in the specific surface area from 6.7 m^2^/g for PGT to 17.2 m^2^/g for PGM.

### 4.4. SEM Analysis

The SEM micrographs (Figure 6a–d) presented shapes of crystallites in sticks and hexagonal structures for PGT and PGW. For PGF, the SEM micrographs exhibited a fibrous aspect. These results may be due to the fact that after washing we removed the soluble impurities and we approached the crystallization form of CaSO_4_. After the modeling of the material (the PGM samples), the SEM micrographs demonstrated that the particles had a regular shape and form. This could have been the result of the recrystallization of CaSO_4_ via the hemi and bihydrate process [32].

**Figure 7:**
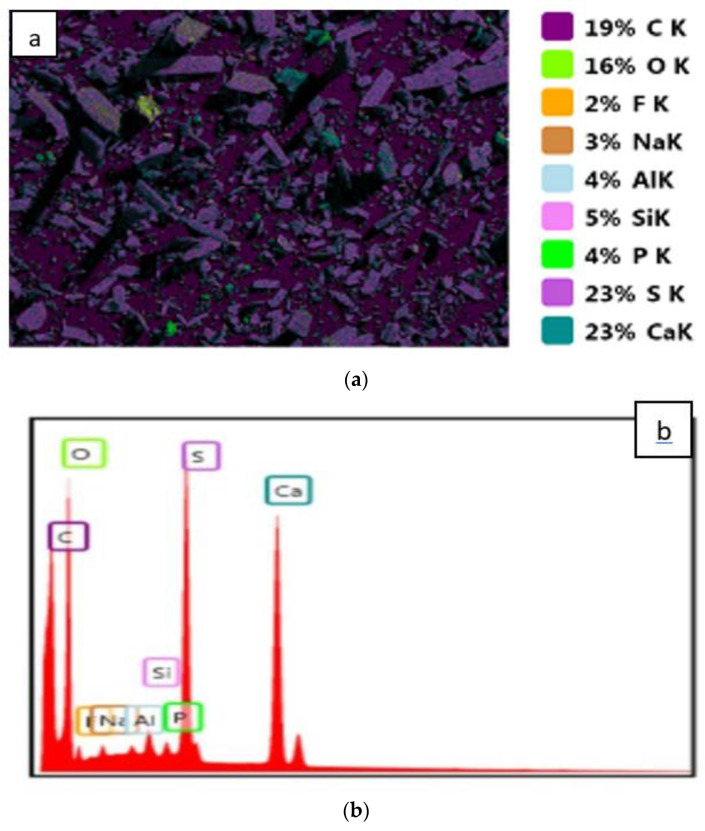
EDS results of PGW: Element map (**a**) and the quantitative ratio of the elements (**b**).

**Figure 8:**
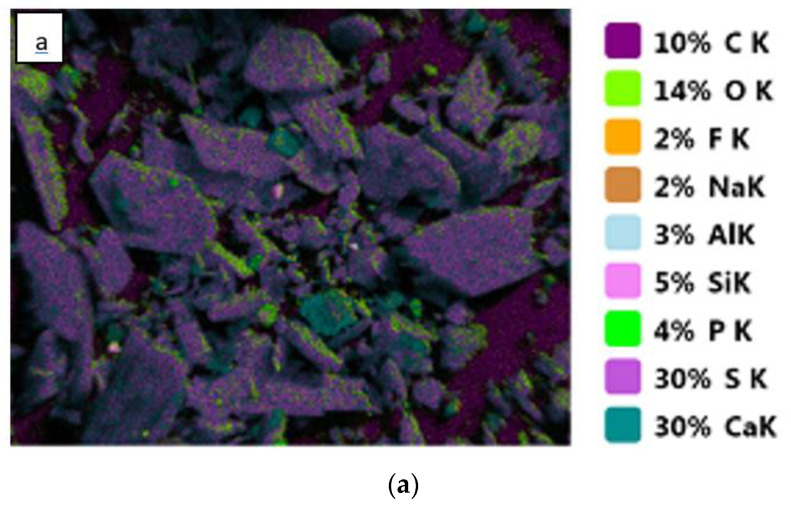

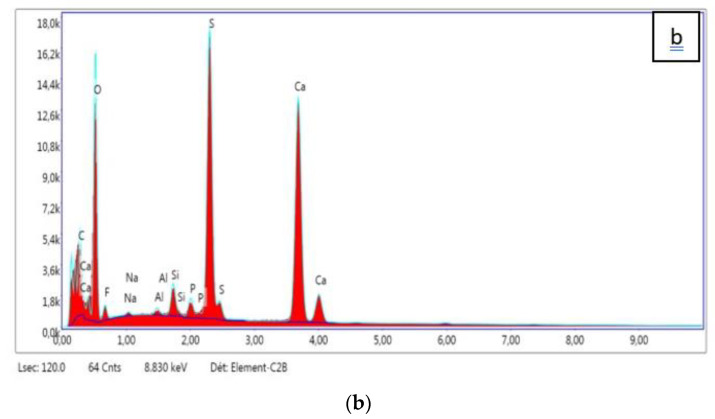
EDS results of PGT: Element map (**a**) and the quantitative ratio of the elements (**b**).

Two samples were analyzed by the EDS technique: PGT and PGW (Figure 7 and Figure 8). The results of the two samples were quite close and confirmed that the majority of the composition was CaSO_4_ with a few impurities such as carbon (C), sodium (Na), fluorine (F), phosphorus (P), silicon (Si) and aluminum (Al).

These maps confirmed the quantitative results of the XRF, which clearly demonstrated that the phosphogypsum samples were formed mainly from gypsum (CaSO_4_). The other elements present such as F, Na, Si and Al that were found with very low quantities could be classified as impurities in the two samples (Table 5).

The carbon contained in the phosphogypsum originated from the organic fraction of the mineral phosphate, which is generally in the form of humic acid [33]. This quantity decreased after the treatment of the sample at 200 °C for 24 h. This decrease could be attributed to the incomplete oxidation of the organic matter.

### 4.5. Thermal Conductivity

The thermal conductivity of the materials was the function of the material density. For the prepared samples, the thermal conductivity was 0.160 W/mK and 0.282 W/mK for the PGM 1/2 (d = 486.4 kg/m^3^) and PGM 1/1 (848.4 kg/m^3^) formulation, respectively (Table 6). These values were similar to those of gypsum mentioned by Ayse et al. [34]. These results confirmed that our prepared samples could be classified as thermal insulation materials.

Gypsum is a hygroscopic salt that has hydration–dehydration equilibrium from low temperatures (between 40 and 70 °C) [35,36]. Its dehydration reaction is endothermic, as shown in Equation (1); therefore, its hydration is exothermic. This equilibrium can participate in the conditioning of the ambient air by a process of water exchange between CaSO_4_.2H_2_O and CaSO_4_.0.5 H_2_O, which is accompanied by a heat exchange; this is the gypsum water pump phenomenon that was verified by DRX and FTIR.

(1) $$\mathrm{CaS}O_{4}.2H_{2}O+heat\;\underset{\leftarrow }{\to }\;\mathrm{CaS}O_{4}.\frac{1}{2}H_{2}O+\frac{2}{3}H_{2}O_{vapor}$$

$$\Delta {H{}^{\circ}}_{\left(25\;{}^{\circ}C\right)}=83.26\;\mathrm{kJ}.{\mathrm{mol}}^{-1}$$

### 4.6. Mechanical Properties

**Figure 9:**
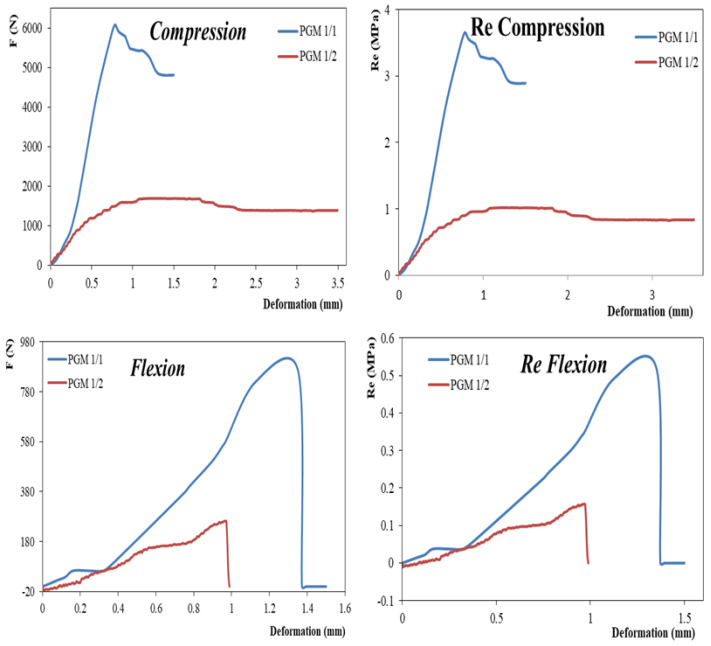
Compressive force, compressive strength, flexion force and flexion strength curves of the prepared materials (PGM 1/1 and 1/2).

Figure 9 shows that the mechanical properties depended on the density. The higher quantity of water used for the preparation considerably decreased the density of the material and caused the fall in the mechanical properties, thus making the material brittle. These results (Table 7) demonstrated that this material derived from phosphogypsum could be used as a building material for non-loadbearing structures or as a plaster or separation plate.

## 5. Conclusions

Based on our results, phosphogypsum can be considered to be a source of gypsum-based material because, in its natural state, it is mainly composed of CaSO_4_ at two degrees of hydration with a few mineral and organic impurities from the phosphate rock. The physicochemical characterization of phosphogypsum demonstrated that the different degrees of hydration that this material possesses allows it to exchange water with the external environment by creating a water pump that helps to condition the ambient air. It should be noted that the mechanical properties of the prepared material PGM 1/1 and PGM 1/2 closely depended on the density. Due to the lower Young’s modulus of these materials, they could be used as construction materials for non-loadbearing structures or as decoration materials. The thermal properties demonstrated that the prepared materials were suitable for insulation in building construction with thermal conductivities lower than 0.3W/mK. It was also concluded that the materials prepared were not suitable for supporting structures.

## Figures and Tables

**Table 1:** Radioactive nuclides in PG.

Nuclide	^238^U	^226^Ra	^228^Ra	^228^Th	^330^Th	^232^Th
Value (Bq/g)	0.05–0.21	0.004–1.48	0.07–0.5	0.001–0.63	0.1–2.9	0.004–0.5

**Table 2:** Average activities of the principal natural radioelements of PG.

	Fresh	10 Years	50 Years
^238^U (Bq/kg)	65.9 ± 1.7	41.2 ± 2.2	35.2 ± 1.9
^232^Th(Bq/kg)	19.7 ± 1.7	16.0 ± 1.4	8.2 ± 1.2
^226^Ra(Bq/kg)	209.4 ± 6.0	209.8 ± 6.9	219.6 ± 6.3

**Table 3:** Chemical composition of the different phosphogypsum samples.

	PGF	PGW	PGT
Fe_2_O_3_ (%)	0.298	0.319	0.366
MgO (%)	1.460	1.489	1.276
Na_2_O (%)	0.586	0.203	0.303
SO_3_ (%)	49.170	50.203	46.489
SiO_2_ (%)	7.638	6.015	6.905
CaO (%)	40.826	41.750	44.302
TiO_2_ (%)	0.0198	0.0193	0.209
CaO/SO_3_	0.830	0.831	0.953

**Table 4:** Textural properties of the PGM and PGT samples.

	BET Surface Area m²/g	Pore Size nm	Pore Volume cm³/g	Nanoparticle Size nm
PGM	17.5	10.9	0.0186	342.6
PGT	6.7	18.1	0.0134	893.8

**Table 5:** Quantitative ratio of the elements in PGW and PGT.

	PGT		PGW	
Element	Weight Ratio %	Atomic Ratio %	Weight Ratio %	Atomic Ratio %
C	9.63	16.82	20.39	32.11
O	40.13	52.62	37.90	44.80
F	2.47	2.72	2.42	2.41
Na	0.34	0.31	0.55	0.45
Al	0.35	0.27	0.37	0.26
Si	1.30	0.97	0.92	0.62
P	0.99	0.67	0.78	0.48
S	16.64	10.88	13.29	7.84
Ca	28.17	14.74	23.38	11.03

**Table 6:** Thermal conductivity and density of PGM 1/2 and 1/1.

Samples	Thermal Conductivity (W/mK)	Density (kg/m^3^)
PGM 1/2	0.160	486.4
PGM 1/1	0.282	848.4

**Table 7:** Mechanical characteristics of the prepared materials.

Density		Compression			Flexion	
		F_max_ N	R_e_ (MPa)	E (MPa)	F_max_ N	R_e_max (MPa)
PGM 1/1	848.4	6083	3.08	253.88	862.5	0.54
PGM 1/2	486.4	1696	1.06	3.14	261.81	0.16
